# A qualitative exploration of potential determinants of accelerated summer weight gain among school-age children: perspectives from parents

**DOI:** 10.1186/s12887-019-1813-z

**Published:** 2019-11-14

**Authors:** Lindsay A. Tanskey, Jeanne P. Goldberg, Kenneth Chui, Aviva Must, Catherine M. Wright, Jennifer M. Sacheck

**Affiliations:** 1 Department of Food, Bioprocessing & Nutrition Sciences, North Carolina State University, Campus Box 7624, Raleigh, NC 27695-7624 USA; 2 Friedman School of Nutrition Science & Policy, Tufts University, 75 Kneeland Street, 8th Floor, Boston, MA 02111 USA; 3 Department of Public Health and Community Medicine, Tufts University School of Medicine, 136 Harrison Avenue, Boston, MA 02111 USA; 4 Department of Exercise and Nutrition Sciences, The George Washington University, 950 New Hampshire Avenue, Washington, DC 20052 USA

**Keywords:** Child obesity, Summer weight gain, Diet, Physical activity, Parents, Qualitative research

## Abstract

**Background:**

There is growing evidence that school children in the United States gain weight more rapidly during the summer than the school year, but few studies have explored the causes of this phenomenon. The goal of this study was to qualitatively explore potential determinants of accelerated summer weight gain by interviewing parents of school-age children.

**Methods:**

Key informant interviews were conducted with parents of third and fourth grade students enrolled in a school-based physical activity intervention in three peri-urban communities in Eastern Massachusetts. A structured interview guide was developed to assess school year and summertime differences in child diet, physical activity, daily routine, and family rules. Interviews were recorded and transcribed verbatim. Transcripts were coded and major themes were identified using thematic analysis.

**Results:**

Summer activities varied substantially by family. Many parents characterized summer as a time with less structure and more relaxed rules, particularly around bedtime and screen use. Parents perceived their child to be more physically active in the summer and reported few barriers to summertime physical activity. Parents reported increases in both positive (increased consumption of fruits and vegetables) and negative (increased consumption of “sweets” and “junk foods”) dietary behaviors. They highlighted several stressors unique to summer, such as the high cost of camps and the need to coordinate childcare and manage children’s time.

**Conclusions:**

Parents perceived their children to be more physically active in the summer and consume more fruits and vegetables than during the school year. However, they also perceived children to consume more energy-dense, nutrient poor foods, engage in more screen time, and have later bedtimes during the summer. These behaviors are important targets for summertime obesity prevention interventions. Large-scale quantitative studies are needed to determine whether these parent perceptions reflect meaningful risk factors for accelerated summer weight gain.

## Background

One-third of children in the United States are overweight or obese [1], and these children face a host of adverse health outcomes [2]. In recent years, summer has emerged as a critical time for obesity prevention. A growing body of evidence suggests that school-age children are susceptible to accelerated rates of weight gain during the summer break compared to the school year [3–6], and that children who are Hispanic or black [7], or overweight [3, 7, 8] are disproportionately affected. This issue has the potential to exacerbate health disparities and counteract school-based obesity prevention efforts [4, 5].

Research is underway to identify the key causes of accelerated summer weight gain so effective policies and interventions can be designed. A model by Baranowski and colleagues suggests that seasonal differences in physical activity, diet, screen media use, and sleep patterns are likely contributors [5]. Few studies have explored school year and summer differences in these factors, and existing findings are mixed. A within-subjects analysis of elementary school children in Massachusetts showed lower fruit and vegetable consumption in the summer, lower engagement in moderate-to-vigorous physical activity (MVPA), and higher engagement in sedentary time than in the school year [6]. Another within-subjects analysis of elementary-aged African American children from low-income households in the Southeastern United States found no significant differences in MVPA but more sedentary time and less light-intensity physical activity (PA) in the summer [9]. Children consumed both fruits and sweets/desserts more frequently during the summer, and slept about 14 min longer. Differences in screen time were more striking: children engaged in 2 more hours of screen time in the summer than the school year. Larger within-subjects studies are needed to identify meaningful differences in obesogenic behaviors from school year to summer among children in the United States. An analysis of National Health and Nutrition Examination Survey (NHANES) data showed poorer dietary patterns and higher levels of physical activity in the summer compared to the school year [10], though different children were surveyed at each time. Another study from Massachusetts reported high levels of sedentary and light activity and poor dietary patterns in summer, though no school year comparison was available [11]. Several studies also show summertime losses in physical fitness in school-age youth [12–15], suggesting that lack of physical activity or increased sedentary time may play a role. However, a study comparing school year and summer total energy expenditure in youth at risk for obesity found no significant difference [16].

Summertime’s relative lack of structure is thought to underlie the unfavorable shifts in obesogenic behaviors responsible for accelerated summer weight gain. Brazendale and colleagues [17] examined this theory by reviewing existing studies that compared children’s obesogenic behaviors on less-structured weekend days (thought to be analogous to summer days) to school days. They found that children generally had less favorable physical activity, sedentary and screen time, sleep, and diet patterns on unstructured days than structured days. The authors acknowledge the heterogeneity of children’s environments and degree of structure during “out-of-school” time, be it the weekend or summer break. Qualitative research can shed light on differences in out-of-school time structure across families and potential impacts on obesogenic behaviors.

Given the equivocal findings and gaps in the literature, research is needed to explore summertime differences in children’s diets, physical activity patterns, and other obesogenic behaviors such as sleep and screen use. Qualitative research with parents on this topic is sparse. A mixed-methods study of low-income minority girls included qualitative parent interviews to explore summertime differences in dietary intake [18]. The parents interviewed perceived their daughters to eat different foods during the summer (including more fruit), to eat more during the summer (such as grazing in front of the television), and to eat at different times in the summer compared to the school year. Additional qualitative research that also explores physical activity, sleep, and screen use can help further illuminate risk factors that emerge when school is not in session and isolate more specific targets for intervention. The goal of this study was to gain a qualitative understanding, through parent interviews, of how children’s diet and physical activity patterns, daily routine (including sleep patterns, screen use, and degree of structure), and family rules differ between the school year and the summer.

## Methods

This research was conducted as part of the FLEX (Fueling Learning through Exercise) Study, a randomized trial to evaluate the effect of two school-based PA programs on PA engagement and academic success [19]. The present study took place over the summer months in six public elementary schools participating in the FLEX Study. The schools were located in three peri-urban communities in eastern Massachusetts. All study procedures and materials were approved by the Tufts University Institutional Review Board.

Parents and caregivers of third and fourth grade students (*n* = 174) enrolled in the first wave of the FLEX Study [19] were eligible to participate in a 30-min telephone interview. Recruitment packets including informed consent documents were sent home with children in Spring 2015. Participants provided written informed consent, and a $30 gift card was provided as an incentive for completing an interview.

The research team created a structured interview guide (Additional file 1) to ensure consistency across the parent interviews. The guide was informed by the aforementioned conceptual model proposed by Baranowski and colleagues [5] of influences on seasonal differences in child BMI change, and by a previous qualitative exploration of summertime health behaviors carried out by members of the research team [11]. Questions were designed to explore school year and summer differences in child diet and PA engagement, the factors governing energy balance. We also included questions about the child’s daily routine (to gain a sense of the degree of structure and sleep patterns during the summer and school year), and family rules related to health behaviors such as screen time, consumption of desserts or “treats,” and enforcement of bedtimes. The guide also included demographic questions to assess parents’ race/ethnicity, marital and employment status, and relationship to the child. The full interview guide is available to readers as a supplement to this article.

Forty-two of the 174 invited parents (24%) agreed to be contacted for a telephone interview, and twenty-eight (67%) of those parents completed one. The remaining parents did not respond to scheduling attempts. An interviewer trained in qualitative research methods (LAT) conducted the 30-min interviews in August and early September. To increase the trustworthiness of the findings, the interviewer engaged in “member checking” throughout the interviews, summarizing the participant’s comments and giving them the opportunity to confirm, correct, and/or expand upon their responses. Interviews were audio recorded and transcribed verbatim by the interviewer for analysis.

A thematic analysis approach was used to identify key themes in the qualitative interviews. An initial coding scheme was developed based on the questions in the structured interview guide. Then, a trained coder (LAT), conducted a first-cycle review of the transcripts, memoing to capture impressions and identify additional themes and codes to emerge from the data. Early findings were discussed within the research team. The coder refined the codebook based on the first-cycle review and group discussion. She then conducted a second-cycle review of the data, independently coding the transcripts using the updated codebook. NVivo qualitative analysis software (Version 11, QSR International, Burlington, MA) was used to efficiently apply codes to the transcripts, and to organize the data for review and synthesis of findings. The findings from this second-cycle review were discussed and the major themes were refined collaboratively by the research team.

## Results

### Demographics

Mothers comprised the majority of interview participants (Table 1). The racial and ethnic diversity of the sample was generally reflective of the schools participating in the study; 32% of parents were non-white. One quarter of participating parents had a child who qualified for free or reduced-price lunch. Thirty-three percent had a child considered overweight or obese, compared to 49% of children in the larger FLEX sample.

**Table 1:** Demographic characteristics of parent interview participants, The FLEX Summer Study, Summer 2015 (*n* = 28)

Variable	Category	n (%)
*Race/ethnicity*	White	19 (68)
	Black	6 (21)
	Hispanic	3 (11)
*Marital status*	Married	25 (89)
	Separated or divorced	3 (11)
*Employment status*	Employed full-time	12 (43)
	Employed part-time	9 (32)
	Not employed	5 (18)
	Student or retired	2 (7)
*Child eligible for free or reduced-price lunch? (n = 26)*	No	19 (73)
	Yes	7 (27)
*Relationship to child*	Mother	25 (89)
	Father	3 (11)

The main themes identified by the research team are summarized below. Illustrative quotes for each theme are presented in Table 2.

**Table 2:** Selected quotations to illustrate key themes from parent interviews (*n* = 28), The FLEX Summer Study, Summer 2015

Theme	Selected Quotations
I. Summer activities vary substantially by family	*“I don’t think there is a typical summer weekday. But in the summer, he has soccer camps and hockey camps …*” *“She wakes up and … because I work during the day, she spends the days with my mother.”* *“She did participate in summer school. That was a six-week program … 1 day a week for 2 h.”* *“Their day-to-day activity will change if they’re going to camp* versus *if they’re staying home. But they’re pretty much scheduled …*” *“Three days a week he goes to camp … and then 2 days during the week he’s with grandparents. And weekends at home with parents.”*
II. Summer means less structure and more autonomy for most children	“*… I kind of just let them be. I don’t pressure them into being on a strict schedule in the summertime. When they’re hungry, they eat … And we just kind of go with the flow … I don’t really make set plans or a set schedule. We just wake up and whatever the day brings, that’s what we end up doing.”* *“During the summertime I kind of just let it … flow. You know, we go to the beach for the day. When we get there, then we just stay, and it’s not as structured. We don’t have to be home at 4:00 to get homework done before any activities or anything like that.”* “*… there’s not the strict structure during the vacation. It’s up to him. He can change activities as he wants.”* *“Because she’s on vacation, and I don’t want to put too much pressure … I want her to be a lot more relaxed and have her own time … she can do her whole schedule. When she wants to go to bed. When she wants to play, she plays, when she want to read, she reads.”*
III. Summer poses few barriers to physical activity	*“I think he’s probably more active during the summer …*. *I think he’s got more time to be active …*” *“I feel like school is like, sit down and shut up, and do your work. You know, you’ve gotta sit there, and he … likes movement. So I feel like he’s able to get more energy out in the summertime …*.” *“I think he’s definitely more active in the summer because of the camp.”* *“Any things that make it more difficult to be active during the summer? No, because I think he is more active during the summer. So … it just makes it easier.”* *“When it was too hot, even though he would want to go out and play, sometimes I would just keep them indoors. I don’t have them go out and play when it’s so hot like that.”* *“Well, the weather, for one … helps tremendously. Even though I gotta say during the winter, we’re out there, too. Then, having no homework. That’s a big thing for her.”*
IV. Summer nutrition can be a double-edged sword	*“Certainly there are better fruits and vegetables available … I think it’s a little bit harder during the winter to get a lot of fresh produce that truly tastes really good.”* *“The pricing is obviously lower in the summer, for people to be able to afford more fruits and vegetables … it’s more available … I think that parents in general … might have more time to actually put the effort into cut up the fruit, and wash it and clean it and already have it set in a Tupperware. And kids can go ahead and open the fridge. They see that and they feel comfortable to kind of help themselves.”* *“I guess because I’m off, I can do more grocery shopping and buy more fresh food. It’s a little bit more convenient … but, it’s also harder. Because no matter where we go somebody else has snacks that they’re sharing so … we try to watch what other people bring.”* *“Well, I think we eat a lot of ice cream in the summer … we go to the beach, they get Italian ice, there’s popsicles. That snacky stuff is definitely more … It becomes more of a common thing in the summer than like a treat, where it wouldn’t be in the winter.”* *“I don’t have that control at Grandma’s, and I know Grandma has a treat drawer.”* *“Yeah, there’s so many fairs, and carnivals, and sidewalk bazaars with junk food and ice cream trucks. And what did we have Saturday? Fried dough!”* “*… it gets hard when there’s like the cookouts, the barbeques … There’s always the burgers, and hot dogs, and … people only have their summer birthday parties or stuff like that.”*
V. Rules are often more negotiable in the summer	*“I teach, so I have summers off. So it’s not a daily routine for us to get up at a certain time … You know, I can let the kids sleep in until they need to, and get whatever sleep they need to. I usually try to get them to bed at a reasonable hour, but if something special is going on … you know, they’ve been able to stay up until … 10:30 at the latest.”* *“In the summertime, like I said, we go out for ice cream more than I care to talk about [laughs]. You know, more than we would during the school year … definitely there are more treats during the summertime.”* *“I think definitely more screen time during the summer, because I am not on top of them. They might be relaxing while I’m trying to do the laundry or do the dishes, and I might lose track of time, so they might get a few extra minutes here and there. So I definitely think during the summer they spend more time on it.”* *“Usually during the summer I am a little bit more lenient [with screen time], because he doesn’t have homework to do … there are still rules, I think it’s just a little bit more lenient …*”
VI. Summer break can be stressful for families	*“I work part time, but you know, money is not flowing, so she comes to work with me. Fortunately, I’m allowed to do that.”* *“It’s harder because they have more free time. There’s more to manage. School does half the day for you during the school year.”* “*… as a working mom … it’s harder right from the bat to figure out what you’re going to do with your kids during the day. Or a working family, with two parents working … the structure of the school year is easier, I think.”* *“Between work and everything like … affording three camps is just not possible for the whole summer for us, so … I have to still work, and find people to watch my kids on the other days, which my parents, or my husband’s parents normally do. But it’s hard, yeah. It’s hard to occupy them and keep them engaged for the whole summer.”*
VII. Families are making an effort to practice healthy behaviors	*“We have fruit available all the time, and they’re allowed to snack on that whenever they want … Vegetables, we try to have them with dinner and if we have one or two, they have to usually finish all of what we give them … They might have a little more during the summer.”* *“A big garden! Right now we have tomatoes, cucumbers, squash, broccoli, green beans, peppers … scallions, onions, herbs. We used to get a vegetable share, where once a week we picked up a giant box full of vegetables. That helps, because everything was new and an adventure to try.”* *“I have three boys, and we have three bowls of fruit on the table at any given moment.”* *“Usually we end our meals with a fruit. We don’t have like a dessert after meals, but we will have like a fruit after meals.”* *“I put vegetables in everything. Like, they just caught me making my meatloaf the other day, and it’s got spinach in it. They’re like, ‘Mom, what are you doing?’ and I’m like, ‘You’ve been eating this your entire life. Get over it.’”* *“I usually try to make them pour into a cup. Like maintain a portion size, not to just walk around with a box of Cheez-its and just … devouring them.”* *“No electronics before bed. Well, we usually watch TV at night, and then usually go up, wind down with some reading, and then go.”*

### I. Summer activities vary substantially by family

Children engaged in a variety of summertime activities, which often depended on the family’s childcare needs. Many attended day camps on weekdays for at least a portion of the summer. Others stayed home with a parent, went to a family member or friend’s home, or accompanied a parent to work. One parent described the day-to-day variability of her child’s summer schedule, stating, “Three days a week he goes to camp … and then two days during the week he’s with grandparents. And weekends at home with parents.” Some children attended overnight camps or traveled for extended periods. Other common activities included free play; spending time outdoors; playing with friends; riding bikes; going to the beach or pool; participating in sports leagues; watching TV; using tablets, computers, and video games; reading; and doing creative and learning activities. For most children, there appears to be significant day-to-day or week-to-week variation in their activities; this is in contrast to the more consistent schedules observed during the school year. As one parent stated, “I don’t think there is a typical summer weekday.”

### II. Summer means less structure and more autonomy for most children

Most parents said their child’s summer routine is less structured than during the school year, and they have more free time and control over their activities. According to one parent, “… there’s not the strict structure during the vacation. It’s up to him. He can change activities as he wants.”

Even children with more structured summer schedules have more free time than during the school year due to lack of homework. As one parent stated, “We don’t have to be home at 4:00 to get homework done before any activities or anything like that.” Parents described this flexibility in both positive and negative contexts. Many said it allows more time for PA. Others said it enables more screen time. Some parents said the reduced structure allows their child more time to eat and promotes eating in response to hunger instead of an arbitrary schedule. Others felt it promotes more snacking due to increased access to food.

### III. Summer poses few barriers to physical activity

Most parents perceived their child to be more physically active during the summer. They attributed this to more free time, better weather, more daylight hours, not having to sit in school or do homework, more time with friends, and opportunities for structured PA at camps. As one parent stated, “I think he’s definitely more active in the summer because of the camp.” Another discussed the increased sedentary time she felt was required of her son during school year, stating, “I feel like school is like, sit down and shut up, and do your work. You know, you’ve gotta sit there, and he … likes movement. So I feel like he’s able to get more energy out in the summertime ….”

Parents said few things interfere with their child’s summertime PA; however, extreme heat and rainy days were mentioned as occasional barriers. Parents described many forms of summer PA, but generally it was characterized by more free play (riding bikes, playing catch, going to the beach or park) while school year PA tends to be more structured (gym class, organized sports, dance lessons).

### IV. Summer nutrition can be a double-edged sword

Most parents said their child consumes more fruits and vegetables (F&V) during the summer. This was attributed to increased access, better quality produce, and lower cost. According to one parent, “The pricing is obviously lower in the summer, for people to be able to afford more fruits and vegetables.” Some parents said the relaxed pace of summer gives them more time to shop for and prepare F&V. Others described growing F&V, visiting farmers markets, and participating in farm shares in the summer months.

However, most parents said their child also consumes more sweets and junk foods during summer. This was attributed to an increased prevalence of special events such as fairs, cookouts, and parties, where less-healthy items are plentiful. One parent described these summertime exposures, stating, “Yeah, there’s so many fairs, and carnivals, and sidewalk bazaars with junk food and ice cream trucks. And what did we have Saturday? Fried dough!”

Many parents described ice cream as a more frequent temptation in summer compared to the school year. Some described a lack of control over their child’s diet in the summer, when many children spend more time away from home and food sharing is common. For example, parents described feeling a lack of control when their child is in the care of a grandparent or when snacks are shared at the beach. One parent said, “I don’t have that control at Grandma’s, and I know Grandma has a treat drawer.”

### V. Rules are often more negotiable in the summer

In general, parents said household rules tend to be more relaxed in the summer. While most families have strict bedtime rules in the school year, many parents said these rules are more flexible during the summer. One parent described this flexibility, stating, “I usually try to get them to bed at a reasonable hour, but if something special is going on … you know, they’ve been able to stay up until … 10:30 at the latest.” Most parents said their child goes to bed later, though some also have the option to sleep in later than they would during the school year. Many parents said they are less vigilant about limiting treats, and screen time rules also tend to be more loosely enforced in the summer. Most said their child engages in more screen time in the summer compared to the school year, when strict rules are maintained to ensure children complete homework. As one parent said, “I think definitely more screen time during the summer, because I am not on top of them.”

### VI. Summer break can be stressful for families

Though many parents described summer as having a more relaxed pace, they also described unique challenges with the potential to influence health behaviors. Arranging for childcare can be difficult, especially in households where parents work full time. Many working parents said they rely on summer camps, which can be expensive, particularly for families with more than one child. One parent described the challenges related to summer childcare, stating, “...affording three camps is just not possible for the whole summer for us, so … I have to still work, and find people to watch my kids on the other days.” Even parents who stay home said summer can be difficult because they have to find ways to fill their children’s time. As one parent stated, “It’s harder because they have more free time. There’s more to manage. School does half the day for you during the school year.”

### VII. Families are making an effort to practice healthy behaviors

Parents described numerous ways they try to help their child develop a healthy lifestyle throughout the entire year. Most said they encourage daily PA and allow their child to choose activities they enjoy. Many described family activities such as going for walks, riding bikes, swimming, playing at the park, and enjoying sports.

Parents showed a good understanding of basic nutrition, and described efforts to promote healthy eating habits within their family. Many said they offer F&V at each meal and make them readily available as snacks. One parent described her practice of incorporating vegetables into her children’s favorite dishes: “I put vegetables in everything. Like, they just caught me making my meatloaf the other day, and it’s got spinach in it.” Several parents said they try to be mindful of portion sizes and limit sweets. As one parent stated, “I usually try to make them pour into a cup. Like maintain a portion size, not to just walk around with a box of Cheez-its and just … devouring them.” Nearly all parents said they ensure their child consumes breakfast daily. Most limit soda consumption and promote water and milk. Nearly all said they eat out infrequently throughout the year, primarily due to its high cost.

## Discussion

These parent insights provide a rich picture of school year and summer differences in child health behaviors likely to contribute to accelerated summer weight gain. The findings underscore the challenges of crafting obesity prevention strategies for the out-of-school environment. The summer landscape is complex, and because children spend their time in so many different types of settings, the issue of summer weight gain is not likely to respond to a “one size fits all” approach. Obesity prevention strategies must be tailored for the different settings where children spend their time when school is out for the summer, addressing summer school, camps, childcare centers, restaurants, parks and community facilities, the built environment, and diverse home environments. Furthermore, it is important for practitioners and policymakers to consider the unique stressors faced by families in the summer, which may play a role in accelerated summer weight gain. For example, the cost of summer childcare and camps paired with loss of free and reduced-price school meals can result in financial strain and increased risk of food insecurity [20]. Increasing access to and awareness of free summer meals through the USDA’s Summer Nutrition Programs is one avenue to promote healthy meals and alleviate financial pressures. Another promising option is the provision of nutrition assistance via electronic benefits transfer during the summer months, to supplement the household food budget when families lose access to school meals. Pilot studies show the potential for this approach to reduce food insecurity [21, 22] and improve nutritional intake in the summer [22].

Nearly all parents perceived their child to be more physically active in the summer. Importantly, parents felt it was easier for their child to engage in physical activity in the summer, because they have more free time, the weather is better, and there are more daylight hours. Quantitative findings comparing school year and summer PA engagement and fitness outcomes are mixed. While some studies have documented declines in fitness over the summer [12–15], the previously mentioned NHANES analysis showed that children surveyed in the summer engaged in slightly more moderate-to-vigorous physical activity than those surveyed during the school year [10]. However, the findings may have been confounded by the fact that NHANES typically samples southern states in the winter and northern states in the summer [23]. A recent study of a later cohort of FLEX participants showed that children engaged in eight fewer minutes of moderate-to-vigorous physical activity and nearly 28 more minutes of sedentary activity in the summer compared to the school year [6]. Another recent within-subjects analysis also found higher levels of sedentary time and lower levels of light-intensity PA in the summer [9]. In light of these conflicting findings, it is important to acknowledge the possibility that parents may not have an accurate perception of their child’s PA engagement at different times of the year. More research using objective measures to identify within-child differences in summer and school-year PA engagement is needed. Given the gap between federal recommendations and child PA engagement in the United States [24], it is likely that even if summer PA engagement is higher than the school year, many children are still falling short of the 60 min daily target.

Our findings suggest that school year and summer differences in dietary behaviors are more complex. Most parents thought their child consumed more F&V in the summer. This aligns with the aforementioned qualitative study including parents of low-income minority girls, in which most parents believed their daughter consumed more fruit in the summer [18]. However, our findings conflict with previously mentioned quantitative studies, including the NHANES analysis, which showed that children surveyed in the summer consumed 0.2 fewer cups of vegetables per day than those surveyed during the school year [10], and the within-subjects FLEX analysis, which showed that students consumed about a half serving less of fruits and vegetables in the summer compared to the school year [6]. Both the present study and the other qualitative study mentioned [18] are based on small, non-representative sub-samples of participants, and it is possible that parents who agreed to participate in interviews and their children differ from those who elected not to participate. The small, within-subjects quantitative study of African American youth aligned with our findings, showing that children consumed both fruit and sugary foods more frequently in the summer [9]. Larger, more representative quantitative studies are needed to clarify how child dietary intake patterns change from the school year to the summer break. It is important to note that though F&V are nutrient-dense and rich in fiber while generally low in calories, there is limited evidence that high F&V intake protects against increased child adiposity [25]. Given that about 95% of school-age American children fall short of F&V recommendations [26], it is likely that summer intakes are suboptimal. Parents also reported an uptick in “treats” and “junk foods” during the summer. This aligns with other studies that showed high summer intakes of energy-dense, nutrient-poor foods [11] and added sugar [10]. It also agrees with evidence that, in younger children, increases in healthy foods are not necessarily accompanied by decreases in unhealthy foods [27].

Decreased structure is another suspected contributor to accelerated summer weight gain [11, 17]. The recently proposed Structured Days Hypothesis posits that the structure conferred on school days limits children’s engagement in obesogenic behaviors [17]. This idea is supported by analyses showing that, in general, children appear to engage in obesogenic behaviors to a greater extent on weekend days than weekdays [17]. Our findings affirm the notion that many children have a less structured routine in the summer, and that this lack of structure goes hand-in-hand with relaxed rules about screen time, bed time, and consumption of “treats” such as ice cream and fried foods, behaviors that have been linked to child obesity [28–31]. Interestingly, parents did ascribe some benefits to this lack of structure, namely increased time to be physically active.

Recently, Moreno and colleagues have proposed an alternative explanation for accelerated summer weight gain, describing how differing environmental cues and social demands in the summer can disrupt children’s circadian rhythms and contribute to unfavorable weight gain [32]. Though our study did not directly explore this concept, our findings that children tended to go to bed later and sleep later in the summertime, engage in more screen time (which would mean increased exposure to artificial light), and experience different family routines and social demands support the possibility that summer could cause greater disruption of circadian rhythms. Further research is needed to explore the contribution of circadian disruption and other bio-behavioral factors to accelerated summer weight gain.

The present study has several limitations. As with any study involving key informant interviews, there is the potential for participation bias. In our study, fewer low-income parents volunteered to give interviews compared to the larger FLEX Study sample, and parents of overweight children were also underrepresented. Our sample consisted only of English-speaking parents, limiting our understanding of child behaviors in households where English is not the primary language. Of particular note, the majority of participants were from a waterfront community with ready access to public beaches and outdoor recreational space. This may have a unique influence on summertime PA engagement and dietary behaviors and may limit generalizability. These qualitative findings can inform the development of quantitative parent surveys to identify factors related to accelerated summer weight gain in larger, more representative samples. Finally, we relied on parent perceptions of their child’s diet and PA patterns, which may not have accurately reflected dietary intake or PA levels, and responses may have been affected by social desirability bias. However, it is a notable strength that our study population includes parents of children who are socioeconomically and racially/ethnically diverse. This study provides a much-needed qualitative understanding of how children’s diets, PA patterns, and daily routines can vary between the school year and the summer.

## Conclusion

Interviews with parents of school-age children suggest several targets for intervention to address excess summer weight gain, including increased consumption of low-nutrient, energy dense foods, shorter sleep duration, and increased screen time. Efforts to alleviate financial strain on families and confer increased structure to children’s days are also likely to improve summer health. The summer landscape is complicated by the diverse settings where children spend time. It is important that summertime obesity prevention efforts reach across these settings. Finally, more quantitative research comparing children’s school year and summer diet, PA engagement, screen time, and sleep patterns is needed to clarify the extent of school year and summer differences and further guide intervention strategies.

## Additional file

**Additional file 1.** The FLEX Summer Health Study Parent Interview Guide: Summer 2015.

## Data Availability

In accordance with the protocol approved by our IRB, the dataset generated and analyzed during the current study are not publicly available. The qualitative nature of the data increase the likelihood that an individual participant may be identified.

## Abbreviations

F&V: Fruit and vegetable
FLEX: The fueling learning through exercise study, an evaluation of school-based physical activity programming
NHANES: National health and nutrition examination survey
PA: Physical activity
