# Supplementary material for: A qualitative exploration of potential determinants of accelerated summer weight gain among school-age children: perspectives from parents
Source: BMC Pediatr. 2019 Nov 14;19:438. doi: 10.1186/s12887-019-1813-z (PMC6854753; doi:10.1186/s12887-019-1813-z)
Supplement: Supplementary file 1 — Additional file 1. The FLEX Summer Health Study Parent Interview Guide: Summer 2015. [file 12887_2019_1813_MOESM1_ESM.pdf]

The FLEX Summer Health Study
Parent Interview Guide
Summer 2015

Objectives:

- To explore differences in children's daily routine, eating habits, and physical activity patterns, and family rules in these areas, between summer vacation and the school year.
- To discuss parent attitudes, norms, and perceived behavioral control regarding their child's nutrition and physical activity during the summer.
- To assess the feasibility of a study assessing children's diet and physical activity patterns during the summer.

Interview Length: 30 minutes

Interview Script

Hello, my name is ____________________ and I am calling on behalf of the FLEX Summer Health Study at Tufts University. Thank you for signing up for a phone interview. Is now still a good time for our 30-minute conversation?

If not, reschedule for another time. If so, proceed below.

You already completed a consent form indicating your willingness to participate in this study, but I would like to quickly review the goals of the study and your role in it to ensure that you would still like to participate.

We are conducting these interviews with parents to learn more about children's behaviors during the summer and the school year. We want to learn more about differences in children's daily routine, diet, and physical activity, and identify ways to help ensure that all children have a healthy summer vacation. We would also like to get your ideas about how we can best conduct a larger study of kids' summer health.

After completing this interview, we will mail you a $30 gift card to Target to thank you for your time and input.

This is considered a "minimal risk" study. I will be audio recording today's conversation so I don't miss any of your comments, but I will not say your full name while the recorder is on, to protect your privacy. The audio files will be stored securely at Tufts until they are transcribed. At that time, any information that would identify you or your child will be removed from the transcripts, and the audio file will be permanently deleted. Your name or your child's name will never be included in any report or publication related to this study.

Participating in this interview will not benefit you directly. It will help us learn more about children's summer health and may lead to improved recommendations, programs, and policies to help ensure that all kids have a healthy summer.

You may choose not to answer any of the questions in this interview – just tell me that you'd prefer not to answer and we'll move on to the next question. Your participation today is voluntary. You may stop participating at any time. There are no right or wrong answers in today's interview.

Would you like to participate today?

If not, thank them for their time and end the call. If so, continue:

May I start the audio recorder?

Discussion Topics:

Parent/Family information (5 minutes)

To start, I'd like to ask a few brief questions about you and your family. Please allow me to read through all the choices before you answer.

1. How many people live in your household? How many of them are under the age of 18?

2. Are you of Hispanic, Latino, or Spanish origin? *(Y / N)*

3. How would you describe your race? You may choose as many responses as needed.
    a. Caucasian/White
    b. Black/African American
    c. Asian
    d. American Indian/Alaska Native
    e. Native Hawaiian or Pacific Islander
    f. Other

4. Which best describes your marital status?
    a. Single, never married
    b. Married or domestic partnership
    c. Widowed
    d. Divorced
    e. Separated

5. Which best describes your employment status?
    a. Work full-time
    b. Work part-time
    c. Not working, but seeking work
    d. Not working and not seeking work
    e. Student
    f. Retired
    g. Other (specify?)________________

6. During our conversation today, we will be referring to your child who just completed 3rd or 4th grade and is participating in the FLEX Study at his or her school. What is your relationship to this child?
    a. Mother
    b. Father
    c. Stepmother
    d. Stepfather
    e. Grandmother
    f. Grandfather
    g. Aunt
    h. Uncle
    i. Guardian
    j. Other

<u>Daily routine, physical activity, and diet in summer vs. the school year</u> (20 minutes)

Daily routine
The next questions are about your child's daily routine during the summer during the summer and the school year.

1. Does your child's daily routine change throughout the summer?

    a. If so, describe how your child's schedule changes from week to week throughout the summer.

2. Where does your child spend weekdays during the summer (i.e. at home; at a friend, family member, or babysitter's home; at camp or summer school; at Boys & Girls Club or YMCA; traveling or on vacation; etc.)?

    a. *Where applicable:* Who is watching him/her in each setting?

3. Where does your child spend weekend days during the summer?

    a. *Where applicable:* Who is watching him/her in each setting?

4. When your child has free time during the summer, how does he or she like to spend it?

5. Thinking about a typical weekday, would you say that your child's summer is more structured or less structured than the school year? Explain.

6. Thinking about a typical weekend day, would you say that your child's summer is more structured or less structured than the school year? Explain.

7. What is a typical bedtime for your child during the school year?

8. What is a typical wake-up time for your child during the school year?

9. What is a typical bedtime for your child during the summer?

10. What is a typical wake-up time for your child during the summer?

11. Tell me about any differences in your child's sleep patterns or bedtime rules between the summer and the school year.

Physical activity

The next questions are about your child's physical activity. Physical activity includes any way that your child actively moves his/her body. It can include light activities such as walking, doing housework, or playing catch; or more vigorous activities such as dancing, running, playing a sport, biking, or swimming.

12. Tell me about the ways your child is physically active during the summer.
    Probe: Think about any organized sports, camps, active hobbies, or play time with friends or at home.

    a. How is this different than what he/she does during the school year?

    b. What things make it easier for your child to be active during the summer?

    c. What things make it more difficult for your child to be active during the summer?

Diet
The next questions are about what your child eats and drinks.

13. Tell me about your child's typical breakfast during the summer.
    Probe: Does your child eat breakfast? If so, what does it include? Where does he/she eat? Who prepares breakfast?

    a. How does this compare to the school year?

14. Tell me about your child's typical lunch during the summer.
    Probe: Does your child eat lunch? If so, what does it include? Where does he/she usually eat? Who prepares lunch?

    a. How does this compare to the school year?

15. Tell me about your child's typical dinner during the summer.
    Probe: Does your child eat dinner? If so, what does it include? Where does he/she usually eat? Who prepares dinner?

    a. How does this compare to the school year?

16. Tell me about your child's typical snacks during the summer.
    Probe: Does your child typically snack? If so, what does it include? When is he/she likely to have snacks? Where does he/she eat? Who prepares or provides snack?

    a. How does this compare to the school year?

17. How do your child's snack and meal patterns differ from a weekday to weekend during the summer?

18. How do your child's snack and meal patterns differ from a weekday to a weekend during the school year?

19. How would you describe a healthful way of eating, in general?
    Probe: What types of foods and drinks should be part of a healthful diet? What types of foods and drinks are not parts of a healthful diet?

20. What things make it easier for your child to eat healthfully during the summer?

21. What things make it more difficult for your child to eat healthfully during the summer?

Household rules

22. Next, I'd like to hear about any rules you have in your household or family. For each area I list, tell me whether your family has any specific rules or practices. Then, tell me how those rules might be different in the summer compared to the school year.
    a. TV or screen time (including use of iPads, iPhones, computers, and video games)

    b. Bedtime

    c. Snacks or treats

    d. Eating out

    e. Staying active (i.e. playing outside, participating in organized sports or lessons)

Parent attitudes, norms, and perceived behavioral control

Next, I'll ask a few questions about parenting during the summer months.

23. In general, how do you feel about the way your child eats during the summer months?
    Probe: Are there any good habits or patterns? Any you'd like to change? Any concerns in comparison to the school year?

a. How sure are you that if you wanted to, you could make positive changes in what your child eats during the summer months? Explain.

b. How do you think other parents feel about the way their children eat in the summer?

24. In general, how do you feel about your child's physical activity during the summer months?
Probe: Are there any good habits or patterns? Any you'd like to change? Any concerns in comparison to the school year?

   a. How sure are you that if you wanted to, you could make positive changes in your child's physical activity during the summer months?

   b. How do you think other parents feel about their child's physical activity in the summer?

25. How are your child safety concerns different in the summer compared to the school year?

26. How are your child health concerns different in the summer compared to the school year?

27. What is easier or harder about parenting during the summer compared to the school year?

28. If you could change anything about your child's summer, what would you change?

<u>Future Summer Research</u> (5 minutes)
We are planning a larger study of children's diet and physical activity over the summer months, and we would love to get your feedback on how to make that study as successful as possible.

29. We would like to collect information about how and where children spend their time during the summer. What might be the best way to gather this information from parents?
    a. Paper logs—like a diary

    b. Emails/online surveys

    c. Phone calls

    d. Other ideas

30. What would be a convenient location for study visits during the summer?
    e. School

    f. Other ideas

31. We would need children and parents to come to two study visits during the summer, each for about 30-45 minutes. Studies like this often offer a gift card or other incentive to encourage people to participate. What types of incentives would appeal to you or your child as a gift for taking part in this type of study?
    g. Gift cards

        i. Preferred stores

    h. Other ideas

SUMMARIZE CONVERSATION.

Do you think that I've got the main ideas from our conversation? Is there anything you would like to add before we finish?

Thank you for your time. I appreciate you sharing your thoughts with me. I will send you your $30 gift card. It will include a slip for you to sign and return to me to indicate that you received it.
